# Modeling and mapping under-nutrition among under-five children in Ethiopia: a Bayesian spatial analysis

**DOI:** 10.3389/fpubh.2025.1553908

**Published:** 2025-05-30

**Authors:** Fekade Getabil Habtewold, Butte Gotu Arero

**Affiliations:** ^1^Department of Mathematics, Kotebe University of Education, Addis Ababa, Ethiopia; ^2^Department of Statistics, Addis Ababa University, Addis Ababa, Ethiopia

**Keywords:** Bayesian, Besag York Mollie, Ethiopia, negative binomial distribution, spatial analysis, under-nutrition, under-five children

## Abstract

Malnutrition remains a critical global challenge, characterized by an imbalance between nutrient requirements and consumption. Under-nutrition, a specific form of malnutrition, results from inadequate intake of essential nutrients and has severe implications for young children, especially in developing countries. This study aims to model under-nutrition cases among children under five in Ethiopia, utilizing Bayesian spatial models to identify effective interventions. Four models were considered: Generalized Linear Model (GLM), Generalized Linear Mixed Models (GLMM), Intrinsic Conditional Autoregressive (ICAR), and Conditional Autoregressive Besag-York-Mollié (CAR BYM) with negative binomial distribution. The rationale for employing multiple models stems from the need to compare performance and accuracy in capturing spatial heterogeneity. The data were obtained from the Ethiopian Demographic and Health Survey 2019. The parameter estimation was carried out using Bayesian Markov Chain Monte Carlo (MCMC) through the brms package in R, which interfaces with Stan for efficient sampling. The models were evaluated based on the Watanabe Akaike Information Criterion (WAIC) and Leave-One-Out (LOO) cross-validation, with CAR BYM emerging as the best-fitting model. Spatial modeling revealed that maternal age, breastfeeding practices, access to clean water and sanitation facilities, cooking practices, maternal education, and wealth status significantly influence the number of under-nutrition cases among children under five in Ethiopia. Specifically, lower maternal education, poorer wealth status, and inadequate access to clean water and sanitation were associated with an increased number of under-nutrition cases, while improved breastfeeding practices, rich wealth status and higher maternal education were associated with decreased number of cases. Regional disparities also played a significant role, with the CAR BYM model effectively identifying high-risk regions such as Somali, Afar, and parts of Oromia, identified as areas requiring targeted intervention.

## 1 Introduction

Malnutrition, particularly under-nutrition, remains one of the most pressing global health challenges, affecting millions and posing significant risks to development and survival. Globally, the burden of under-nutrition is staggering, with approximately 7.7% of children under five (around 52 million) affected by wasting and 22.9% (154.8 million) stunted, indicating chronic under-nutrition. These forms of malnutrition contribute to long-term adverse health outcomes, including impaired cognitive development and increased mortality rates, with nearly 45% of deaths among children under five linked to under-nutrition (1, 2). The burden is disproportionately distributed, with 94% of stunted children residing in Asia and Africa, underscoring the need for targeted interventions in these regions (2).

In sub-Saharan Africa (SSA), under-nutrition continues to be a major issue due to socioeconomic challenges, food insecurity, and limited access to healthcare (2). Ethiopia, one of the countries most affected, exhibits alarmingly high rates of malnutrition. According to the Ethiopian Demographic and Health Survey (EDHS), 37% of children under five (approximately 2.8 million) are stunted, while 21% (around 1.6 million) experience wasting (3, 4). These statistics highlight chronic under-nutrition and its profound impact on physical and cognitive development. Additionally, regional disparities within Ethiopia, such as higher malnutrition rates in Tigray compared to Addis Ababa, emphasize the necessity of region-specific analyses and interventions (3).

Efforts to assess and address under-nutrition often utilize anthropometric indicators such as stunting, wasting, and underweight. While socioeconomic status, maternal education, health practices, and environmental factors have been identified as significant determinants of childhood malnutrition, their effects vary widely. Socioeconomic status significantly influences child nutrition, with children from poorer households facing a higher risk of under-nutrition due to limited access to nutritious foods, healthcare, and sanitation (5–7). Maternal education is another critical factor, influencing nutritional knowledge and child-feeding practices, with studies demonstrating that increased maternal education correlates with improved child health outcomes (8, 9). Health-related practices, such as exclusive breastfeeding and dietary diversity, are essential but often challenging to implement effectively (10, 11). Environmental factors, including access to clean water and sanitation, further exacerbate under-nutrition, particularly in regions with inadequate infrastructure (12, 13).

Despite various interventions implemented in Ethiopia to combat under-nutrition, such as community-based nutrition programs, health extension services, and food security initiatives, the persistence of high under-nutrition rates suggests that these efforts may not fully address the complex interplay of spatial and contextual factors. Understanding the spatial heterogeneity of under-nutrition prevalence is crucial for designing more effective and targeted interventions (14). Previous studies in Ethiopia have explored the determinants of under-nutrition using various models, including spatial regression, multilevel analysis, generalized linear models, geo-additive mixed models, and geographically weighted regression (4, 15–18). However, these approaches may risk model misspecification and inaccurate estimates when heterogeneity or extreme response values are present, particularly if linear relationships are assumed.

Traditional statistical models, such as Generalized Linear Models (GLM) and Generalized Linear Mixed Models (GLMM), often fall short in capturing the spatial complexities inherent in under-nutrition data, especially when geographic, sociocultural, and economic conditions exhibit significant variation across regions. Count data frequently display over-dispersion or under-dispersion, which violates the assumption of independent observations (19). While GLMs and GLMMs are not inherently spatial models, they can be adapted for spatial data analysis by incorporating spatial covariates or structured random effects. For instance, GLMMs can include random intercepts to account for spatial clustering or dependencies, addressing some spatial variation within the dataset (20). However, these adaptations do not enable GLMs and GLMMs to directly model spatial autocorrelation which is better addressed by specialized spatial models like Conditional Autoregressive (CAR) and Besag-York-Mollié (BYM) models (21). These models are specifically designed to handle spatial dependencies and heterogeneity, offering greater robustness for spatial analysis. While GLMMs can partially account for spatial dependencies through spatially structured random effects, their primary focus remains on clustered or multilevel data, making them less optimal for modeling the unique characteristics of spatial autocorrelation. Dedicated spatial models, therefore, provide a more suitable framework for analyzing the complex spatial relationships inherent in under-nutrition data.

To address these challenges, spatial models, such as the Bayesian Conditional Autoregressive (CAR) model, specifically the Besag-York-Mollié (BYM) model and its more recent and computationally efficient refinement, are more appropriate (20). These models accommodate both fixed and random effects, account for unobserved heterogeneity and spatial autocorrelation, and facilitate the assessment and prediction of disease risk in specific geographical areas (21–25).

This study investigates under-nutrition in Ethiopian children under five, aiming to identify key covariates and map regional risk. To achieve this, we employed and compared four modeling approaches: Generalized Linear Models (GLM), Generalized Linear Mixed Models (GLMM), Intrinsic Conditional Autoregressive (ICAR) models, and Conditional Autoregressive Besag-York-Mollié (CAR BYM) models. The GLM and GLMM served as baseline models, while the ICAR and BYM models were used to assess spatial patterns and improve risk mapping accuracy.

## 2 Methods

### 2.1 Data and study design

This study utilizes data from the 2019 Ethiopian Demographic and Health Survey (EDHS), a comprehensive and nationally representative survey that provides extensive information on the demographic and health characteristics of households across Ethiopia. The dataset includes critical insights into various aspects, such as fertility and family planning behaviors, child mortality, nutritional status of children, utilization of maternal and child health services, and knowledge of HIV/AIDS and sexually transmitted infections (STIs).

The EDHS was conducted across 11 geographic areas, encompassing 9 regions and 2 city administrations. The surveys employed a stratified two-stage cluster sampling approach. In the first stage, clusters were selected, followed by the selection of households in the second stage. Clusters were categorized based on the respondents’ residence (rural vs. urban) and the specific districts (Woredas) from which they were drawn. Each district (Woreda) is further subdivided into sub-districts (Kebeles), which are then divided into census enumeration areas (EAs). Selected enumeration areas also included spatial information, providing latitude and longitude coordinates, which enhances the analysis of geographic variations in health outcomes. This robust sampling design ensures that the findings are representative and can effectively inform public health strategies in Ethiopia (3).

### 2.2 Study variables

The primary outcome variable in this study is child under-nutrition, specifically assessed through the presence of stunting and/or wasting in children within surveyed households. A child is considered undernourished if they exhibit either stunting or wasting, or both, as defined by the World Health Organization Child Growth Standards (26). Stunting is defined as a height-for-age z-score below −2 standard deviations (SD) of the WHO Child Growth Standards median, while wasting is defined as a weight-for-height z-score below −2 SD of the same median. The analysis will focus on the prevalence of under-nutrition within the surveyed population, using a binary classification to determine whether a child is undernourished (yes/no) based on these criteria.

To investigate the association with under-nutrition, the following covariates were included in the models: mother’s age (years), mass media exposure, breastfeeding status, mother’s body mass index, source of drinking water, type of toilet facility, type of cooking materials, place of delivery, residence type (urban or rural), mother’s educational status, household wealth index, sex of household head, cluster identifier, and region. The description and measurements of these variables are presented in Table 1. The potential risk factors include the selected socio-demographic, environmental, and maternal health covariates, which were chosen based on findings from existing literature (4, 6, 9, 11, 13, 14, 17).

**Table 1:** Description of study variables in the model.

Variables	Description	Measurements
Stunting	Stunting Height-for-age Z-score	Z-score < −2 indicates stunting; Z-score ≥ − 2 indicates normal growth.
Wasting	Wasting Weight-for-height Z score	Z-score < −2 indicates wasting; Z-score ≥ − 2 indicates normal weight-for-height
Mothers age (years)	Age of the mother in years.	Mother’s age 15–49 years
Mass media	Exposure to media, reflecting the mother’s access to information.	No (ref) Yes
Breastfeed	Child is being breast fed.	No (ref) Yes
Mother’s body mass index	Body Mass Index of the mother, indicating nutritional status.	As a ration weight/ (height)^2^
Source of drinking water	Source of drinking water (e.g., piped, well, surface).	Improved (ref) Unimproved
Type of toilet facility	Category of toilet facility (e.g., improved or unimproved).	improved (ref) unimproved
Type of cooking materials	Cooking practices or methods used in the household.	Modern (ref) Traditional
Place of delivery	Place of delivery (e.g., home, health facility).	Home/otherplaces—ref Hospital/ Health center
Residence	Residence type (urban or rural).	Rural (ref) Urban
Mothers educational status	Level of educational attainment of mother	No education (ref) Primary Secondary Higher
Household wealth index	Household wealth quintiles (a measure of socio-economic status)	Poorest—ref Poorer Middle Richer Richest
Sex of household head	Gender of the household.	Female—ref Male
Cluster	Cluster identifier for the survey sampling.	Spatial (1, 2, …, 305)
Region	Region (State where mothers live)	Spatial ϵ {1, 2, 3, …, 11}

### 2.3 Statistical data analysis

Exploratory Spatial Data Analysis (ESDA) was used to examine spatial autocorrelation, patterns, clusters, outliers, and heterogeneity of under-nutrition across Ethiopia. Bayesian modeling was employed to make inference in hierarchical models variety of distributions, such as negative binomial (27).

Various modeling approaches including Generalized Linear Models (GLM), Generalized Linear Mixed Models (GLMM), Intrinsic Conditional Autoregressive (ICAR) models, and Conditional Autoregressive Besag-York-Mollie (CAR BYM) models were applied and comparisons were made. By employing these advanced statistical methods, this research aims to provide a robust analysis of under-nutrition cases among children in Ethiopia, ultimately contributing to improved understanding and targeted interventions in public health.

#### 2.3.1 Spatial dependence and spatial heterogeneity analysis

Spatial autocorrelation, also referred to as spatial dependence, is the evaluation of how similar or dissimilar two attribute values are when they are located close to each other in space. It is also a way to describe the presence or absence of variations in a variable. In this particular study, we employed spatial autocorrelation analyses to identify the geographic distribution of under nutrition cases among children below the age of five in Ethiopia. To assess the spatial patterns of under nutrition, we utilized specific spatial statistical indices, namely Moran’s I and the Getis-Ord (Gi*) indices.

The importance of Moran’s I statistic lies in its ability to establish the association between the values of a variable at one location and those at other locations. This statistical measure considers both the value and location of a variable, providing valuable insights into the relationship between the variable and its surroundings (28). Moran’s I ranges from −1 to 1, with a score of zero indicating no clustering. A positive score, on the other hand, suggests a spatial concentration of similar values. The formula to calculate Moran’s I is as follows (Equation 1):


(1) $$I=\frac{n\sum_{i}\sum_{j}W_{\mathrm{ij}}(Y_{i}-\bar{Y})(Y_{j}-\bar{Y})}{(\sum_{i}\sum_{j}W_{\mathrm{ij}}){\left((Y_{j}-\bar{Y})\right)}^{2}}$$

Where, n is the total number of spatial units (clusters), $Y_{i}$ and $Y_{j}$ are the values of the response variable (under-nutrition) for clusters 𝑖 and 𝑗, respectively, and $W_{\mathrm{ij}}\;$is the weight used for the comparisons made between locations i and j and the elements of the weight matrixW (see Section 4.2.3 for definition of weight matrix).

As one of the objectives of this study, the spatial patterns of undernourished and its underlying factors across the country were identified. It is believed that this plays a pivotal role in identifying areas that displayed either significantly high or low levels of under nutrition in Ethiopia. To achieve this, the study employed the application of Getis-Ord local statistics, which provided valuable insights into the extent of the malnutrition issue (29).

The formula to calculate Getis-Ord local statistics is as follows (Equation 2):


(2) $${G_{i}}^{*}=\frac{\sum_{j=1}^{n}W_{\mathrm{ij}}Y_{j}-\bar{Y}\sum_{j=1}^{n}W_{\mathrm{ij}}}{S\sqrt{\frac{n\sum_{j=1}^{n}{W_{\mathrm{ij}}}^{2}-{\left(\sum_{j=1}^{n}W_{\mathrm{ij}}\right)}^{2}}{n-1}}}$$

Where, n is the total number of spatial units (clusters), $Y_{j}$ is the number of undernourished at location j, W is a weight matrix between locations i and j and $S=\sqrt{\frac{\sum_{j=1}^{n}{Y_{j}}^{2}}{n}}-{\left(\bar{Y}\right)}^{2}$.

The fact that the level of spatial dependency may vary significantly across the space suggests that the capacity to detect and pinpoint spatial heterogeneity is more desirable (30). The family of Moran indices, still, did not have the ability to discriminate between hot spots and cold spots areas. The Gi* index therefore came in handy to solve the problem of identifying hotspots and cold spots.

The expected Getis-ord (G) for a threshold distance d, is defined as (Equation 3):


(3) $$E(G(d))=\frac{W_{*}}{n(n-1)}$$

Where $W_{*}$ is the sum of weights for all pairs of locations $W_{*}=\sum_{i}\sum_{j}W_{\mathrm{ij}}$. Assuming normal distribution, the variance of G and the Z statistic are defined as (Equation 4):


(4) $$\mathrm{Var}(G(d))=E(G^{2})-{\left(E(G)\right)}^{2}$$

Thus (Equation 5),


(5) $$Z(G(d))=\frac{G(d)-E(G(d))}{S.E(G(d))}$$

Where $S.E(G(d))=\sqrt{\mathrm{Var}(G(d))}$ represents the standard error of the Getis-Ord statistic *G*(*d*), which quantifies the variability or uncertainty associated with the calculated G(d) for a given distance d.

In order to determine the spatial arrangement tendencies, we calculated the mean center and the standard distance. The mean center, as employed in this study, refers to the latitude and longitude coordinates of all characteristics encompassed by the study. Its computation is suitable for monitoring alterations in the spatial distribution of these characteristics as well as their associations. Lastly, the *p* value is computed to assess the significance of clustering related to under-five under nutrition, and the Z-score is utilized to test its statistical significance (29).

#### 2.3.2 Methods for detecting over-dispersion

Over-dispersion occurs when the observed variance in count data exceeds the variance predicted by a statistical model, such as Poisson regression. This phenomenon is common in count data, where the assumption of equal mean and variance inherent in the Poisson distribution may not hold. If over-dispersion is present and not addressed, it can lead to inefficient estimates, biased standard errors, and incorrect inferences, such as misleading *p*-values. Detecting over-dispersion is crucial, as it indicates that the chosen model may not adequately capture the underlying data structure. One effective method for detecting over-dispersion is Deviance Analysis. This statistical technique assesses the goodness of fit of a model by comparing it to a saturated model, which perfectly aligns with the data. Deviance Analysis helps identify whether the variability in the data exceeds what the model assumes (31).

The deviance D of the fitted model is calculated using the following formula (Equation 6):


(6) $$D=-2(\log(L_{\text{null}})-\log(L_{\text{model}}))$$

In this equation $L_{\text{null}}$ represents the likelihood of the null model (intercept-only model), and $L_{\text{model}}$ denotes the likelihood of the fitted model.

To assess over-dispersion, the deviance D is compared to the degrees of freedom df of the model. After calculating the deviance D statistics, divide by their respective degrees of freedom to get the over-dispersion ratios defined as follows.

##### 2.3.2.1 Over-dispersion ratio

This ratio is calculated by dividing the deviance by the degrees of freedom. A ratio significantly greater than one indicates over-dispersion, while a ratio close to one suggests an appropriate model fit. This approach is vital for ensuring that the statistical model chosen provides a reliable representation of the data, ultimately supporting more accurate inference and predictions (31).

### 2.4 Statistical model specification

This study employs a comprehensive statistical methodology to analyze the risk of under nutrition among children under five in Ethiopia. The analysis utilizes several approaches, including General Linear Models (GLMs), Generalized Linear Mixed Models (GLMMs), Intrinsic Conditional Autoregressive (ICAR) models, and Conditional Autoregressive Besag-York-Mollié (CAR BYM) models. The CAR models are particularly well-suited for geospatial data on under nutrition, enabling the capture of spatial dependencies.

#### 2.4.1 Generalized linear models

Generalized Linear Models (GLMs) extend traditional linear models to allow for response variables that have error distribution models other than a normal distribution. They consist of three components:

- **Random Component:** Specifies the distribution of the response variable (e.g., Poisson, binomial, negative binomial).
- **Systematic Component:** Represents the linear predictor, which is a linear combination of the explanatory variables.
- **Link Function:** Connects the random and systematic components, allowing the model to handle various types of data.

In the context of under nutrition, a GLM can model the count of undernourished individuals as a function of various predictors. The Poisson and Negative Binomial distributions are especially useful for modeling count data (31).

##### 2.4.1.1 Poisson regression

Poisson regression is a type of Generalized Linear Model (GLM) used to model the relationship between a response variable that follows a Poisson distribution and predictor variables. The Poisson distribution is the most common distribution for count data, suitable when the counts are independent and the mean is equal to the variance.

The general formulation for GLM with a Poisson distribution is as follows (Equation 7):


$$Y_{k}\sim \text{Poisson}(\mu_{k})$$

(7) $$\log(\mu_{k})=\beta^{T}X_{k}$$

In this equation: $Y_{k}$ is the *k-th* response variable that follows a Poisson distribution, $\mu_{k}$ is the mean of the Poisson distribution, $X_{k}$ is the vector of predictors and β is the vector of parameters to be estimated. The logarithm function serves as the link function to relate the linear predictors to the response variable.

##### 2.4.1.2 Negative binomial regression

Negative Binomial Regression is a specialized form of Generalized Linear Model (GLM) specifically designed for modeling count data that exhibits over-dispersion where the variance exceeds the mean. This method is particularly useful when the response variable follows a negative binomial distribution. Negative Binomial Regression provides a flexible framework for analyzing count data, allowing researchers to model complex relationships between the response variable and predictors while accounting for over-dispersion. This makes it a valuable tool in various fields, including epidemiology, ecology, and other sciences.

The general formulation for GLM with a negative binomial distribution can be expressed as follows (Equation 8):


(8) $$Y_{k}\sim \mathrm{NB}(\mu_{k},\gamma )$$

$$\log(\mu_{k})=\beta^{T}X_{k}$$

Where, $Y_{k}$ is the i-th response variable that follows a negative binomial distribution, $\mu_{k}$ is the mean of the distribution, $X_{k}$ is the vector of predictors,β is the vector of parameters to be estimated, γ is the dispersion parameter and log(.) functions as the link of the negative binomial distribution.

In summary, Generalized Linear Models (GLMs) were employed to establish the baseline relationship between various predictors and the count of under-nourished children. Specifically, Poisson and Negative Binomial regression were utilized to accommodate the count nature of the data, with the Negative Binomial model accounting for over-dispersion. These models allowed for the identification of significant predictors associated with under-nutrition, providing a foundation for more complex spatial analyses.

#### 2.4.2 Generalized linear mixed models

Generalized Linear Mixed Models (GLMMs) extend Generalized Linear Models (GLMs) by incorporating both fixed effects (predictors that apply to the entire population) and random effects (predictors that vary across groups or clusters). This allows for the modeling of hierarchical or clustered data structures, which are common in geographical studies (32). In this research, GLMMs can account for variability among different regions by including random effects for regional factors. This is important for accurately estimating the prevalence of under nutrition while considering the inherent correlation within regional data.

In this model, we assume that the response variable $Y={\left(Y_{1},Y_{2},\ldots Y_{n}\right)}^{T}$ is a univariate vector representing the count of under-nutrition cases at location k. Each $Y_{k}$ follows a Negative Binomial distribution. The spatial pattern of the responses was modeled by the covariate matrix $X=({X_{1}}^{T},{X_{2}}^{T},\ldots ,{X_{n}}^{T})$ and the structured spatial random effect $\Phi =(\Phi_{1},\Phi_{2},\ldots ,\Phi_{n}\;)$. The covariate at the location 𝑘 is expressed as $X_{k}={\left(1,x_{1k},x_{2k},\ldots ,x_{\mathrm{pk}}\right)}^{T}$, where $x_{\mathrm{jk}}$ denotes the values of the *j-th* predictor at location k with j=1,2,…,p and k=1,2,…,n. The Conditional Autoregressive (CAR) model can be formulated to incorporate these elements effectively, allowing for a better understanding of the spatial dynamics influencing under-nutrition rates. The CAR model can be formulated as follows (Equation 9):


(9) $$Y_{k}|\mu_{k}\sim \mathrm{NB}(\mu_{k},\gamma )$$

(10) $$\log(\mu_{k})=\beta^{T}X_{k}+\Phi_{k}$$

where $\mu_{k}$ is the expected count, and $\beta ={\left(\beta_{0},\beta_{1},\ldots ,\beta_{p}\right)}^{T}$represents the fixed effect coefficients (Equation 10).

In this context, structured refers to the spatial random effects $\phi_{k}$ that account for the correlation of observations based on their locations.

##### 2.4.2.1 Structured spatial random effects

These effects capture the underlying spatial correlation in the data. In many cases, observations that are geographically close to each other are more likely to be similar than those that are further apart. The structured random effects take this spatial dependency into account, allowing the model to better fit the data by recognizing patterns related to location. By including structured spatial random effects, the model can account for unobserved heterogeneity across locations, helping to improve predictions and inference regarding the counts of under-nutrition cases.

In summary, Generalized Linear Mixed Models (GLMMs) were used to incorporate the hierarchical structure of the data, specifically accounting for regional variability. By including random effects for regional factors, GLMMs provided a more accurate estimation of under-nutrition prevalence, considering the inherent correlation within regional data. The use of Conditional Autoregressive (CAR) models within the GLMM framework allowed for the modeling of spatial dependencies, enhancing the understanding of spatial dynamics influencing under-nutrition rates.

#### 2.4.3 Intrinsic conditional autoregressive models

The Intrinsic Conditional Autoregressive (ICAR) model is specialized form of the Conditional Autoregressive (CAR) model that simplifies the modeling structure by assuming that the mean of the random effects is zero. This characteristic makes the ICAR model particularly useful for focusing on differences between locations rather than absolute values. ICAR models are designed to analyze spatially correlated data, wherein the value of a variable at a given location is influenced by the values at neighboring locations (33). The intrinsic property of the model ensures that the sum of the spatial random effects equals zero, preventing any bias from being introduced into the analysis.

The outcome variable of this study (undernourished cases) $Y={\left(Y_{1},Y_{2},\ldots Y_{n}\right)}^{T}$ follows a negative binomial family of distribution. The spatial pattern of the responses was modeled by the covariate matrix $X=({X_{1}}^{T},{X_{2}}^{T},\ldots ,{X_{n}}^{T})$ and the spatial random effect $\Phi =(\Phi_{1},\Phi_{2},\ldots ,\Phi_{k})$. The covariate at the location 𝑘 is expressed as $X_{k}={\left(1,x_{1k},x_{2k},\ldots ,x_{\mathrm{pk}}\right)}^{T}$ and $\beta ={\left(\beta_{0},\beta_{1},\ldots ,\beta_{p}\right)}^{T}$ is the vector of regression parameters. Then, the general formulation of Bayesian conditional autoregressive (CAR) is an extension of a generalized linear model and is given by (Equation 11):


(11) $$Y_{k}|\mu_{k}\sim \mathrm{NB}(\mu_{k},\gamma ),k=1,2,\ldots ,n$$

(12) $$\log(\mu_{k})=\beta^{T}X_{k}+\Phi_{k}$$

In the ICAR model, the spatial random effects are expressed as (Equation 12):


(13) $$\Phi_{k}=\sum_{j\in N(k)}W_{\mathrm{kj}}(\Phi_{j}-\Phi_{k})+\in_{k}$$

where $W_{\mathrm{kj}}$ represents the weights assigned to the neighboring locations, and $\in_{k}\sim N(0,\sigma^{2})$ are normally distributed error term (Equation 13).

ICAR models are particularly well-suited for geospatial data concerning under-nutrition, as they effectively capture spatial dependencies. For instance, if one region exhibits high rates of under-nutrition, neighboring regions are likely to be affected as well, potentially due to shared environmental or socioeconomic factors. This ability to model such interdependencies enhances the understanding of spatial dynamics in public health research.

To account for uncertainty in covariate effects on undernourished cases, we use a hierarchical prior for the regression coefficients $\beta_{j}$, assuming a normal distribution centered at zero with variance ${\tau_{\beta }}^{2}$, which itself follows an Inverse-Gamma distribution:

$\beta_{j}\sim N(0,{\tau_{\beta }}^{2})$, where ${\tau_{\beta }}^{2}\sim \text{Inverse}-\text{Gamma}\;(a,b).$

This approach allows data-driven estimation of variance, offering flexibility in shrinkage and robustness to scaling differences. Choosing small values for a and b (e.g., 0.001) results in a weakly informative prior, ensuring inference is primarily guided by the observed data rather than strong assumptions. By adapting to covariate variability, this hierarchical prior enhances model performance in spatial analyses of under-nutrition (25).

The commonly used CAR models for spatial autocorrelation include the ICAR and the Besag-York-Mollié (BYM) models, both proposed by Besag et al. (25), as well as the Leroux model (24) and the Stern-Cressie model (23). The prior for the random effect is assumed to follow a Gaussian Markov random field (GMRF), which can be expressed as (Equation 14):


(14) $$\Phi \sim N(0,\tau^{2}Q^{-1})$$

Where 𝑸 is a precision matrix that may be singular (intrinsic model)

This matrix controls the spatial autocorrelation structure of the random-effects and is based on a non-negative symmetric 𝑛 × 𝑛 neighborhood or weight matrix W. The specification requires that the correlations between geographically neighboring areas (i.e., when $W_{\mathrm{jk}}=1$) are enforced for $\left(\phi_{j},\phi_{k}\right)$ pairs, while random effects for non-contiguous areas are considered to be conditionally independent when the values of the remaining random effects are given (34).

Spatial weight matrices are essential for modeling spatial relationships in various analyses, particularly in Bayesian Conditional Autoregressive (CAR) models. Below are the methods for constructing the spatial weight matrix W using different weighting schemes, including queen contiguity, exponential weight, and inverse distance weight.

The queen contiguity method assigns weights based on adjacency, considering both edge and corner neighbors:


$$W_{\mathrm{ij}}=\{\begin{array}{c} 1\;\text{if}\;i\;\text{and}\;j\;\mathrm{are}\;\text{neighbors}\\ \;0\;\text{if}\;i\;\text{and}\;j\;\mathrm{are}\;\text{not neighbors}\; \end{array}$$

The value of $W_{\mathrm{ij}}$ is the value of the element in the adjacency matrix corresponding for the 𝑖-th area and 𝑗-th area. The value of one is given if the 𝑖-th area is adjacent to the 𝑗-th area, while a value of null is given if the 𝑖-th area is not adjacent to the 𝑗- th area. In our case the area is Region (11 regions of Ethiopia).

Exponential weighting assigns weights that decrease exponentially with distance distance (35).

$W_{\mathrm{ij}}=\exp(-d_{\mathrm{ij}})$, where $d_{\mathrm{ij}}$ being the distance between location i and j and given by:


$$d_{\mathrm{ij}}=\sqrt{{\left({\text{latitude}}_{i}-{\text{latitude}}_{j}\right)}^{2}+{\left({\text{longitude}}_{i}-{\text{longitude}}_{j}\right)}^{2}}$$

Inverse distance weighting gives higher weights to closer areas (36):

$W_{\mathrm{ij}}={d_{\mathrm{ij}}}^{-1}$, where $d_{\mathrm{ij}}$ is distance between areas i and j.

Standardizing a weight matrix in all approaches, can enhance the robustness and interpretability of spatial analyses, particularly in contexts where weights vary significantly across regions.

To select an appropriate spatial weight matrix, we computed Moran’s Index using various weighting schemes, including Queen Contiguity, Exponential weighting, and Inverse distance weighting. By comparing the Moran’s Index values across these schemes, we identified which matrix effectively captured the spatial relationships inherent in our data. A higher and statistically significant Moran’s Index indicated that the chosen weight matrix aligned well with the spatial dependencies in our analysis, guiding us toward a more robust model for this study.

#### 2.4.4 Conditional autoregressive Besag-York-Molli model

The simplest CAR prior is the Intrinsic Autoregressive (IAR) prior, which was proposed by Besag et al. (25). It is characterized by its full conditional distribution, which can be expressed as follows (Equation 15):


(15) $$\Phi_{k}|\Phi_{-k},W,\tau^{2}\sim N(\frac{\sum_{j=1}^{n}W_{\mathrm{kj}}\Phi_{j}}{\sum_{j=1}^{n}W_{\mathrm{kj}}},\frac{\tau^{2}}{\sum_{j=1}^{n}W_{\mathrm{kj}}})$$

(16) $$\tau^{2}\sim \text{Inverse}-\text{Gamma}\;(a,b)\;$$

In this set up, the conditional expectation is the average of the random effects in neighboring areas$\Phi_{j}$, where $W_{\mathrm{kj}}$ represents the weight (or adjacency) between locations. The variance of the conditional distribution is inversely proportional to the sum of the weights of neighboring locations, scaled by the parameter $\tau^{2}$ (Equation 16). This suggests that areas with more neighbors (higher weights) will have lower conditional variance in their random effects, effectively highlighting how the random effect at a specific location is influenced by its neighbors while accounting for spatial dependence.

In summary, the Intrinsic Conditional Autoregressive (ICAR) model was utilized to specifically address the spatial dependencies inherent in the under-nutrition data. By assuming a zero mean for the random effects, the ICAR model focused on the relative differences between regions, effectively capturing how neighboring areas influence each other. This model allowed for the analysis of spatially correlated data, wherein the prevalence of under-nutrition in one area is influenced by neighboring areas, thus enhancing the understanding of spatial dynamics in public health research. The use of the ICAR model was essential for identifying spatial patterns and dependencies that could not be captured by non-spatial models.

The Conditional Autoregressive Besag-York-Molli (CAR BYM) model combines the concepts of CAR models with a structured random effects approach (25). It divides the spatial random effects into two components, one that captures the overall spatial trend and another that accounts for local variations. This model is advantageous in the study of under nutrition as it allows for nuanced spatial analysis, helping to identify regions that are not only affected by general trends but also by local conditions. The Conditional Autoregressive (CAR) Besag-York-Mollié (BYM) model is particularly effective for analyzing count data, which often exhibit over-dispersion where the variance exceeds the mean. Count data typically represent non-negative integers, such as the number of occurrences of an event (e.g., undernourished cases in a specific area). To address this over-dispersion, the CAR BYM model employs a negative binomial distribution rather than a Poisson distribution. The negative binomial distribution introduces an additional parameter that provides flexibility in modeling the variance, making it well-suited for count data.

The outcome variable $Y={\left(Y_{1},Y_{2},\ldots Y_{n}\right)}^{T}$ follows a negative binomial family of distribution, which is suitable for count data with over-dispersion and the covariate matrix $X=({X_{1}}^{T},{X_{2}}^{T},\ldots ,{X_{n}}^{T})$. Specifically, the model can be expressed as (Equation 17):


(17) $$Y_{k}\sim \mathrm{NB}(\mu_{k},\gamma )$$

(18) $$\mu_{k}=\beta^{T}X_{k}+\Phi_{k}+u_{k}$$

where $\mu_{k}$ is the mean of the negative binomial distribution, γ is the dispersion parameter, $\Phi_{k}$ is the conditional autoregressive random effect (structured component) and $u_{k}$ is the spatial unstructured residuals (Equation 18).

In the context of statistical modeling, particularly in hierarchical or mixed models, “unstructured” typically refers to a type of residual or random effect that does not have a specific pattern or structure imposed on it. Unstructured residuals are treated as independent and identically distributed ($u_{k}\sim N(0,{\tau_{u}}^{2})$) random variables. This means that they do not follow a specific correlation structure, unlike structured random effects that account for spatial or temporal correlations.

Besag et al. (25) assumed that the two random effects are independent and require a specification of independent priors. The prior distribution model for the spatially unstructured $u_{k}$ are assumed to follow a normal distribution: $u_{k}\sim N(0,{\tau_{u}}^{2})$, where $\;{\tau_{u}}^{2}\sim \text{Inverse}-\text{Gamma}\;(a,b)$ and the prior distribution for the CAR model as defined above in Equation 15.

Moreover, both the Leroux and Stern & Cressie modeling approaches can be utilized to model varying strengths of spatial autocorrelation. The Leroux model’s key feature is its ability to model spatial autocorrelation that varies across different areas, with the parameter ρ weighting neighboring effects for a more flexible representation of spatial relationships, while using a single set of random effects to simplify the modeling process and capture complex spatial structures. The Stern and Cressie (23) model accommodates complex autocorrelation structures and allows for tailored spatial effects, making it suitable for hierarchical Bayesian modeling that provides a nuanced understanding of data through multiple levels of random effects. Alternative CAR priors for modeling varying strengths of spatial autocorrelation, using only a single set of random effects proposed by Leroux et al. (24) is given by (Equation 19):


(19) $$\Phi_{k}|\Phi_{-k}\sim N(\frac{\rho \sum_{j=1}^{n}W_{\mathrm{kj}}\Phi_{j}}{\rho \sum_{j=1}^{n}W_{\mathrm{kj}}+1-\rho },\frac{\tau^{2}}{\rho \sum_{j=1}^{n}W_{\mathrm{kj}}+1-\rho })$$

The model by Stern and Cressie (24) is (Equation 20)


(20) $$\Phi_{k}|\Phi_{-k}\sim N(\frac{\rho \sum_{j=1}^{n}W_{\mathrm{kj}}\Phi_{j}}{\rho \sum_{j=1}^{n}W_{\mathrm{kj}}+1-\rho },\frac{\tau^{2}}{\rho \sum_{j=1}^{n}W_{\mathrm{kj}}+1-\rho })$$

The mean of the conditional distribution is a weighted average of the random effects $\Phi_{j}$ at neighboring locations, scaled by the parameter ρ. This parameter adjusts the influence of neighboring random effects based on the degree of spatial correlation. The variance of the conditional distribution is inversely proportional to the sum of the weights of neighboring locations, adjusted by ρ. This indicates that as the spatial correlation increases (higher ρ), the conditional variance decreases, reflecting greater certainty about the random effect at location k.

The prior distribution for ρ is considered to be uniformly distributed between 0 and 1, denoted as ρ~unif(0,1). The Conditional Autoregressive Besag-York-Mollié (CAR BYM) model is widely used in spatial statistics for analyzing data with spatial correlations.

In summary, the Conditional Autoregressive Besag-York-Molli (CAR BYM) model was employed to provide a comprehensive analysis of under-nutrition prevalence, accounting for both structured and unstructured spatial effects. By combining CAR models with a structured random effects approach and utilizing a negative binomial distribution to address over-dispersion, the CAR BYM model allowed for a nuanced spatial analysis. This model effectively captured the overall spatial trends as well as the local variations in under-nutrition, enabling the identification of regions affected by both general trends and local conditions. The CAR BYM model provided a robust framework for analyzing count data with spatial correlations, offering valuable insights into the spatial dynamics of under-nutrition in Ethiopia.

### 2.5 Parameter estimation

In this study, we employed Bayesian methods for parameter estimation, utilizing the brms package in R, which interfaces with Stan a powerful platform for statistical modeling. The Bayesian approach allows us to incorporate prior information and obtain a full posterior distribution for our parameters of interest.

The full posterior distribution in Bayesian statistics is obtained by combining the likelihood of the data given the parameters with the prior distributions of those parameters, as expressed by Bayes’ theorem (37, 38) (Equation 21).

This relationship can be formulated as:


(21) $$P(\theta |Y)=\frac{P(Y|\theta )*P(\theta )}{P(Y)}$$

Where, P(θ∣Y) is the posterior distribution of the parameters θ and given the data Y, P(Y∣θ) is the likelihood of the data given the parameters, P(θ) is the prior distribution of the parameters and P(Y) is the marginal likelihood of the data, which acts as a normalizing constant.

The posterior distribution can be obtained mathematically using the following steps.

#### 2.5.1 Step 1: specify the likelihood function

The likelihood of the data Y given the parameters γ in a negative binomial model can be expressed mathematically as follows (39) (Equation 22):


(22) $$\begin{array}{l} L(Y|X,\Phi ,u,\gamma )\\ =\prod_{k=1}^{n}\frac{\Gamma (Y_{k}+\gamma^{-1})}{\Gamma (Y_{k}+1)\;\Gamma (\gamma^{-1})}\;{\left(\frac{\mu_{k}}{\mu_{k}+\gamma^{-1}}\right)}^{Y_{k}}{\left(\frac{\gamma^{-1}}{\mu_{k}+\gamma^{-1}}\right)}^{\gamma^{-1}}\; \end{array}$$

#### 2.5.2 Step 2: specify priors

The prior distribution for *β* with inverse variance (precision, $\tau_{\beta }=\frac{1}{\;{\sigma_{\beta }}^{2}}$) (40) (Equation 23):


(23) $$P(\beta )=\prod_{j=0}^{p-1}\frac{1}{\sqrt{2\pi \;{\tau_{\beta_{j}}}^{2}}}\exp(-\frac{{\beta_{j}}^{2}}{2\;{\tau_{\beta_{j}}}^{2}})$$

For the unstructured random effects $u_{k}$ (25) (Equation 24):


(24) $$\begin{array}{c} P(u)=\prod_{k=1}^{n}[\frac{1}{\sqrt{2\pi \;{\tau_{u}}^{2}}}\exp(-\frac{{u_{k}}^{2}}{2\;{\tau_{u}}^{2}})]\\ ={\left(\frac{1}{2\pi \;{\tau_{u}}^{2}}\right)}^{\frac{n}{2}}\exp(-\frac{1}{2\;{\tau_{u}}^{2}}\sum_{k=1}^{n}{u_{k}}^{2})\; \end{array}$$

The prior distribution for the structured spatial effect $\Phi_{k}$ in Bayesian spatial models is typically derived from a Conditional Autoregressive (CAR) or Intrinsic Conditional Autoregressive (ICAR) framework. The probability density function (PDF) of the structured spatial effect $\Phi =(\Phi_{1},\Phi_{2},\ldots ,\Phi_{k})$ can be expressed as follows (Equation 25):


(25) $$p(\Phi |W,{\tau_{\Phi }}^{2})\propto \exp(-\frac{1}{2\;{\tau_{\Phi }}^{2}}\sum_{k=1}^{n}\sum_{j\in N(k)}W_{\mathrm{kj}}{\left(\Phi_{k}-\Phi_{j}\right)}^{2})$$

The combined probability density function of the prior distributions can therefore be expressed as Robert (37) (Equation 26):


(26) p(β)∗p(Φ)∗p(u)

##### 2.5.2.1 Step 3: determine the posterior

By substituting the expressions for the likelihood and priors, the full posterior distribution can be written as (37, 38) (Equation 27):


(27) $$\begin{array}{l} p(\beta ,\Phi ,u,{\tau_{\beta }}^{2},{\tau_{\Phi }}^{2},{\tau_{u}}^{2}|Y,X,\gamma )\propto p(Y,X,\gamma |\beta ,\Phi ,u,{\tau_{\beta }}^{2},{\tau_{\Phi }}^{2},{\tau_{u}}^{2})\\ {}*p(\beta )*p(\Phi )*p(u) \end{array}$$

This leads to (Equation 28):

$\begin{array}{l} p(\beta ,\Phi ,u,{\tau_{\beta }}^{2},{\tau_{\Phi }}^{2},{\tau_{u}}^{2}|Y,X,\gamma )\propto \prod_{k=1}^{n}\frac{\Gamma (Y_{k}+\gamma^{-1})}{\Gamma (Y_{k}+1)\Gamma (\gamma^{-1})}\\ \;{\left(\frac{\mu_{k}}{\mu_{k}+\gamma^{-1}}\right)}^{Y_{k}}{\left(\frac{\gamma^{-1}}{\mu_{k}+\gamma^{-1}}\right)}^{\gamma^{-1}}*\prod_{j=0}^{p-1}\frac{1}{\sqrt{2\pi \;{\tau_{\beta_{j}}}^{2}}}\exp(-\frac{{\beta_{j}}^{2}}{2\;{\tau_{\beta_{j}}}^{2}})* \end{array}$


(28) $$\begin{array}{l} {}*\exp(-\frac{1}{2\;{\tau_{\Phi }}^{2}}\sum_{k=1}^{n}\sum_{j\in N(k)}W_{\mathrm{kj}}{\left(\Phi_{k}-\Phi_{j}\right)}^{2})*{\left(\frac{1}{2\pi \;{\tau_{u}}^{2}}\right)}^{\frac{n}{2}}\\ \exp(-\frac{1}{2\;{\tau_{u}}^{2}}\sum_{k=1}^{n}{u_{k}}^{2}) \end{array}$$

##### 2.5.2.2 Hyper prior distribution

Besag et al. (25) postulated that the two random effects are independent, necessitating the specification of separate prior distributions. Accordingly, the hyper prior distributions for the precision parameters are defined as follows:

${\tau_{\Phi }}^{2}\sim \text{Inverse}-\text{Gamma}\;(1,0.0005)$,$\;{\tau_{u}}^{2}\sim \text{Inverse}-\text{Gamma}\;(1,0.0005)$ and ${\tau_{\beta }}^{2}\sim \text{Inverse}-\text{Gamma}\;(1,0.0001)$. The variance component parameters $\;{\tau_{u}}^{2}$and $\;{\tau_{\Phi }}^{2}$ control the variability of $u_{k}$ and $\Phi_{k}$ respectively as stated in Lawson et al. (41). A non-informative normal distribution is assumed on the fixed effect, i.e., $\beta ∼N(0,{\sigma_{\beta }}^{2})$.

The parameter estimation was executed using Bayesian Markov Chain Monte Carlo (MCMC) through the brms package in R, which interfaces with Stan for efficient sampling. After defining the model and specifying prior distributions for the parameters, MCMC employed the No-U-Turn Sampler (NUTS) to explore the posterior distribution by iteratively updating parameter values based on their likelihood given the data. Model performance was evaluated through convergence diagnostics, such as trace plots, and posterior predictive checks, which compared simulated data from the posterior distribution to actual observations, ensuring that the model adequately captured the underlying patterns in the data. This comprehensive formulation provides a robust framework for parameter estimation within the BYM model, allowing for effective analysis of under nutrition data while accounting for both structured and unstructured spatial effects (42).

In summary, a Bayesian framework was employed for parameter estimation, utilizing the brms package in R and Stan for MCMC sampling. This approach allowed for the incorporation of prior information and the estimation of the full posterior distribution for the model parameters. Specifically, the Besag-York-Molli (BYM) model was used to capture both structured and unstructured spatial effects in the under-nutrition data. The use of Bayesian methods, including the specification of likelihood functions and prior distributions, provided a robust and flexible approach to parameter estimation, allowing for the analysis of complex spatial dependencies. The evaluation of model performance through convergence diagnostics and posterior predictive checks ensured the reliability and validity of the results. The Bayesian parameter estimation was conducted using Markov Chain Monte Carlo (MCMC) methods, specifically implemented through the `brms` package (R 4.3.2) in R (27), which interfaces with Stan. For each model, we ran four independent MCMC chains to assess convergence. Each chain consisted of 2000 iterations, with the first 1,000 iterations discarded as burn-in. This allowed the chains to reach their stationary distribution, ensuring that the samples drawn were representative of the posterior distribution.

### 2.6 Model diagnostics

Model diagnostics are crucial for assessing the fit and validity of a Bayesian model, particularly when using Markov Chain Monte Carlo (MCMC) methods (42). Here are key components of model diagnostics for the BYM model estimated in this study using the brms package.

#### 2.6.1 Convergence diagnostics

Visual inspection of the trace plots for each parameter helps determine if the MCMC chains have mixed well and converged to the posterior distribution. Ideally, the traces should look like a “fuzzy caterpillar” without apparent trends or patterns.

This statistic compares the variance within each chain to the variance between chains. Values close to one suggest good convergence, while values greater than one indicate that more iterations may be needed.

The ESS quantifies the number of independent samples drawn from the posterior distribution. A higher ESS indicates better mixing of the chains. Generally, an ESS of 400 or more per parameter is considered adequate.

This involves generating simulated data based on the model’s posterior distribution and comparing it to the observed data. Visualizations, such as histograms or density plots, can help assess whether the model adequately captures the data distribution and underlying patterns.

#### 2.6.2 Model fit statistics

When evaluating the fit and comparing models, two commonly used techniques are the Widely Applicable Information Criterion (WAIC) and Leave-One-Out Cross-Validation (LOO). These techniques offer valuable insights into model adequacy, facilitating informed decisions during model selection processes (43).

WAIC is a versatile statistical criterion used to assess model fit and make comparisons between different models. It is particularly valuable in Bayesian statistics for model selection and evaluation. WAIC provides a measure of the trade-off between goodness of fit and model complexity, aiding in the selection of the most appropriate model. Lower WAIC values indicate better model performance in terms of predictive accuracy and goodness of fit (43).

LOO is a technique used to evaluate the predictive performance of models by iteratively leaving out individual data points and assessing how well the model predicts these left-out points (43).

## 3 Results

The analysis of under-nutrition data was conducted using the Negative Binomial Conditional Autoregressive (CAR) method with the brms package in R 4.3.2 (27). The process began with data exploration, followed by calculating Moran’s Index to assess spatial autocorrelation in the response variable. Over-dispersion was checked by examining the deviance and Pearson chi-squared values relative to their degrees of freedom. A Spatial Weight Matrix W was constructed using the Queen’s contiguity weighting. Prior distributions for hyperparameters in the Intrinsic CAR (ICAR) and Besag-York-Mollié (BYM) models were specified. Model selection criteria included the Leave-One-Out Cross-Validation (LOO) and Widely Applicable Information Criterion (WAIC). The optimal model’s relative risk was determined by using the brms package in R by fitting a Negative Binomial CAR model.

### 3.1 Data exploration

**Figure 1:**
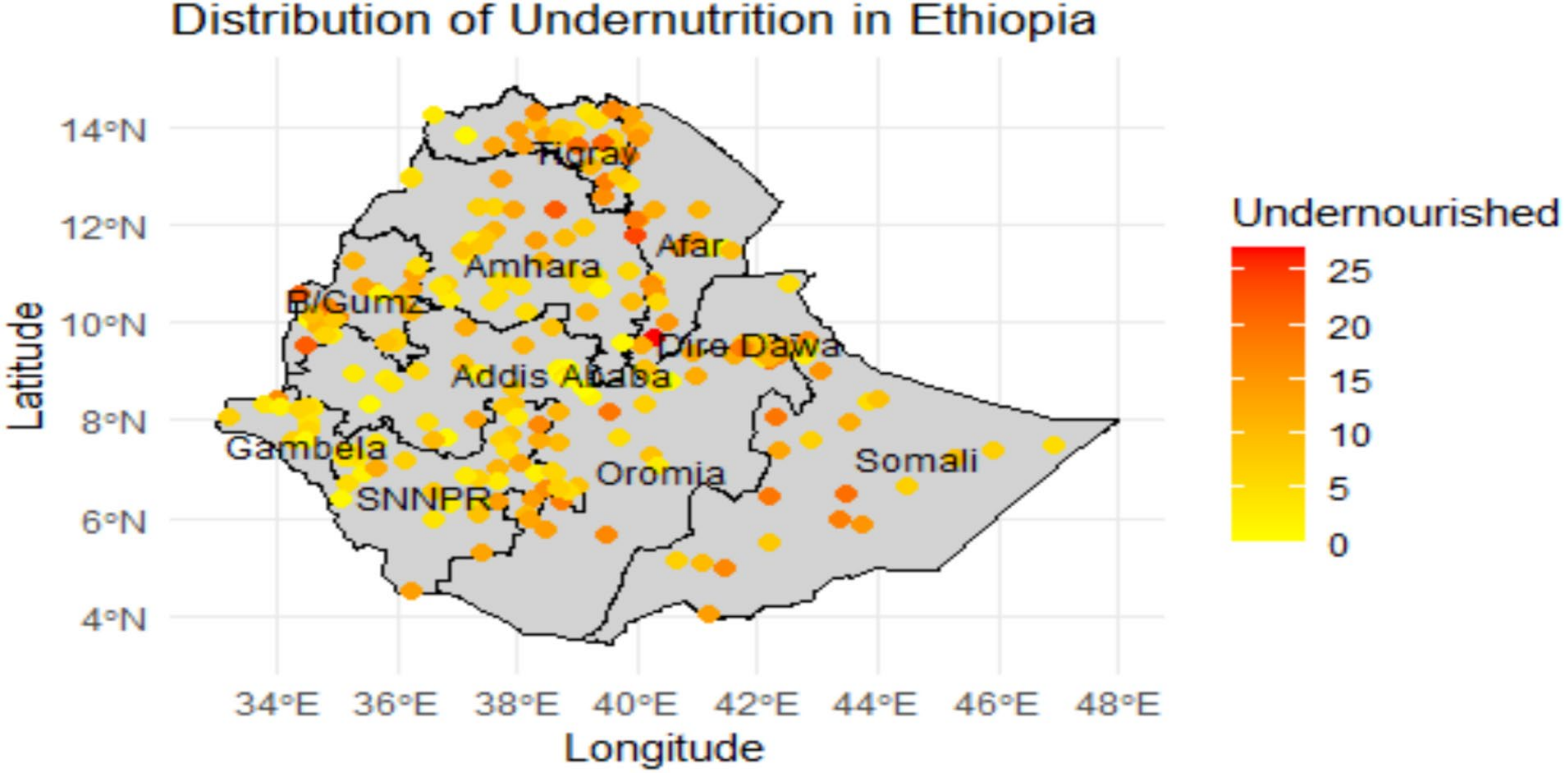
Spatial distribution of number of undernourished under-five children (defined as children with wasting, stunting, or both) by study clusters and Regional level in Ethiopia 2019.

The distribution map of under-nutrition presented in Figure 1 illustrates the spatial distribution of under-nutrition among children under five years old in Ethiopia, revealing significant regional disparities in nutritional status. In this study, ‘undernourished’ is defined as children who exhibit either wasting (low weight-for-height), stunting (low height-for-age), or both, based on the WHO child growth standards. Each dot on the map represents the number of undernourished children under-five years in a given cluster (enumeration area). Dots in red and orange indicate a high prevalence of under-nutrition, while areas shaded with yellow indicate a low prevalence. Notably, areas in the eastern and southern regions, such as Somali, show a higher number of under-nourished children, particularly in rural areas. This suggests that these regions face more severe nutrition challenges compared to urban areas like Addis Ababa. The geographical representation allows for a clear comparison between regions, underscoring the urgent need for targeted public health initiatives in areas most affected by under-nutrition. The data for this map is obtained from the Ethiopian Demographic and Health Survey 2019. Overall, the map serves as an important tool for understanding child nutritional status disparities in Ethiopia, guiding efforts to improve nutritional outcomes across the country.

#### 3.1.1 Exploratory data analysis for under-nutrition amongst the under-five children in Ethiopia

The results of the over-dispersion tests presented in Table 2 below, for both the Poisson and Negative Binomial models suggest important insights into their fit for the data. For the Poisson model, the Pearson Chi-Squared statistic is 17,333.19, with an over-dispersion ratio of 3.34, indicating significant over-dispersion, as this value is considerably greater than one. The deviance for the Poisson model is 18,943.58, with a similar over-dispersion ratio of 3.65, further confirming that the model does not adequately capture the variability in the data.

**Table 2:** Over-dispersion testing results.

Test	Poisson	Negative binomial
Pearson Chi-Squared	17333.19	4418.35
df(degree of freedom)	5,192	5,192
Over-dispersion ratio (Pearson)	3.34	0.85
Deviance	18943.58	5744.13
Over-dispersion ratio (Deviance)	3.65	1.11

The reference point of one for the over-dispersion ratio is critical because it reflects the Poisson model’s assumption that the mean and variance of the data are equal. A ratio of one indicates that the observed variance aligns with this assumption, suggesting a good fit. Values greater than one indicate that the observed variance exceeds the mean, signifying over-dispersion and suggesting that the Poisson model may not be appropriate for the data. This underlines the need for alternative models, such as the Negative Binomial, which can better account for the increased variability.

In contrast, the Negative Binomial model demonstrates a much better fit, with a Pearson Chi-Squared value of 4,418.35 and an over-dispersion ratio of 0.85, suggesting that there is no over-dispersion present. The deviance for the Negative Binomial model stands at 5,744.13, yielding an over-dispersion ratio of 1.11, which is only slightly above one, indicating a minor potential for over-dispersion. Overall, the Negative Binomial model is preferable for this dataset, effectively accommodating the variability without the significant over-dispersion observed in the Poisson model.

In this study, we utilized the Queen Contiguity matrix, which identifies relationships between regions based on shared edges and corners. This matrix includes diagonal neighbors, allowing for a more comprehensive analysis compared to other matrices. Given the complex nature of under-nutrition, it is influenced by interconnected factors—environmental, social, and economic—that often extend beyond simple boundary lines. These factors can “spill over” into neighboring areas, including those that share a vertex. The Queen Contiguity matrix effectively accounts for spatial dependencies that go beyond direct edge-sharing.

Table 3 presents the Moran’s Index values for under-nutrition using Queen Contiguity weight matrices. The Queen’s Contiguity method demonstrated considerable spatial autocorrelation, with a Moran’s Index of 0.3437, indicating significant clustering of high under-nutrition rates, as evidenced by a *p*-value of 0.0000. These findings underscored the significant spatial clustering of under-nutrition, highlighting the need for targeted interventions in affected regions. As a result, the study employed the Queen’s Contiguity weight matrix for further analysis, focusing on describing under-nutrition regions of Ethiopia.

**Table 3:** Moran’s index value of under nutrition.

Weighted matrix	Moran’s index	E(I)	Var(I)	*p*-value
Queen contiguity	0.3437	−0.0033	0.0014	0.0000

**Figure 2:**
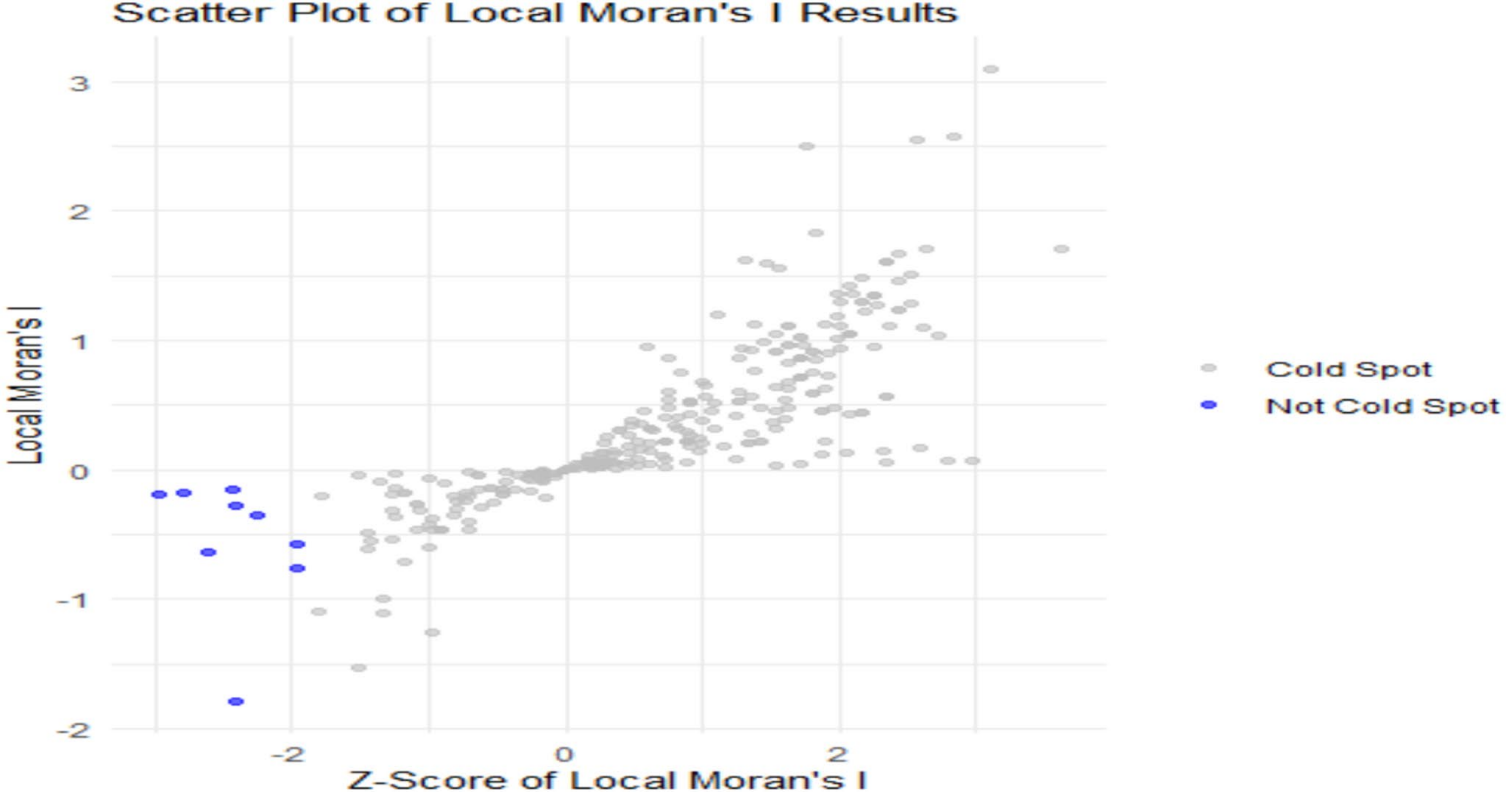
Scatter plot of Local Moran’s I results.

The scatter plot in Figure 2 illustrates the Local Moran’s I results, indicating the relationship between Z-scores of Local Moran’s I and their corresponding values. The x-axis represents the Z-scores, which showed how much local values differed from the average, while the y-axis displayed the Local Moran’s I values. The blue points indicate “cold spots,” representing areas with low levels of under-nutrition that clustered with other low values, while areas with high numbers of undernourished children “hot spots” are indicated with gray points. In contrast, the gray points, which were not categorized as cold spots, primarily clustered around zero or exhibited positive Local Moran’s I values, suggesting they did not show significant patterns of low values. While most points were centered near zero, some blue points were noticeably in the negative geographical location, highlighting specific areas with concentrated low values. These cold spots suggested locations that might have required targeted interventions or further investigation related to under-nutrition. Overall, the scatter plot effectively showed the spatial distribution of under-nutrition, identifying areas that needed more focus.

**Figure 3:**
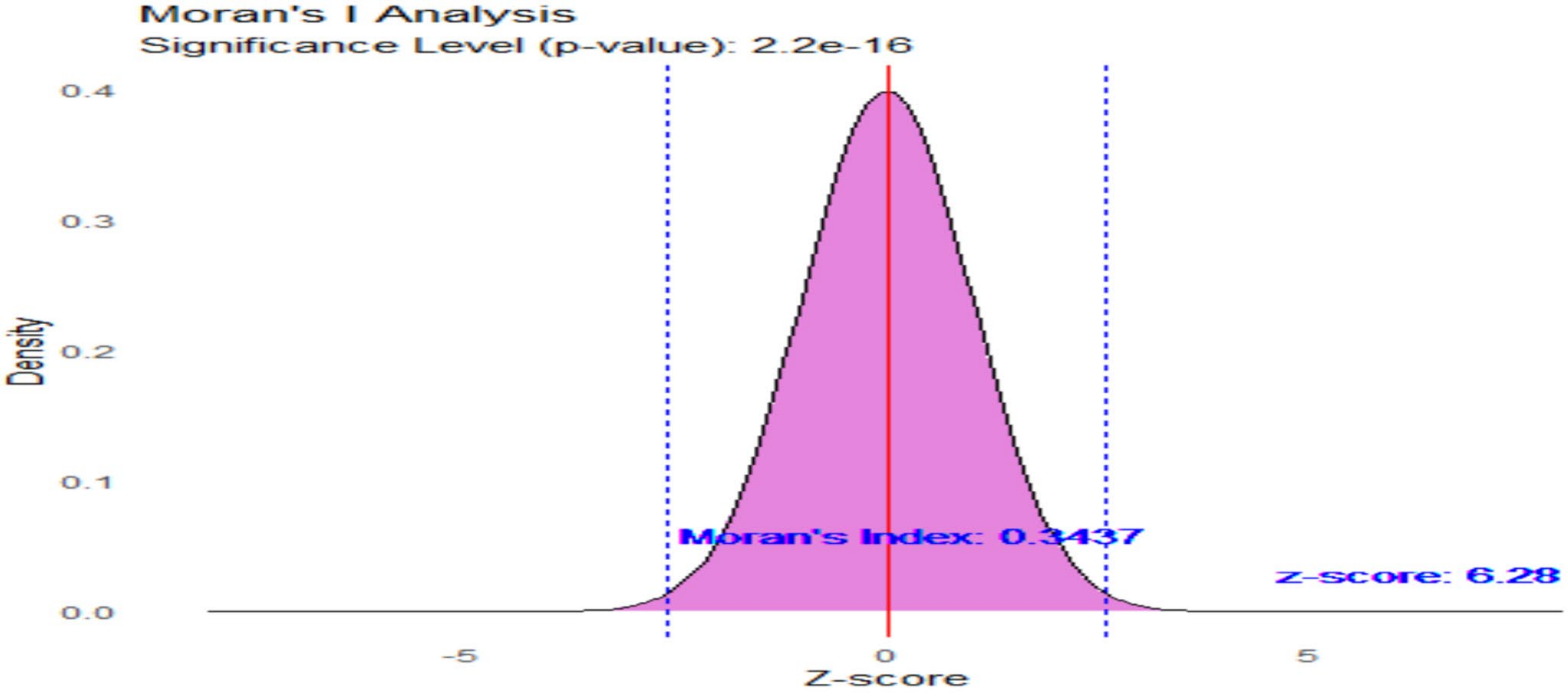
Moran’s index of spatial autocorrelation for under nutrition in Ethiopia.

Figure 3 presented the “Moran’s I Analysis” plot, which illustrated the distribution of Z-scores related to spatial autocorrelation in the context of under-nutrition. The bell-shaped curve, typical of a normal distribution, reflected the density of Z-scores under the null hypothesis of no spatial autocorrelation. The extremely low *p*-value of 2.2e-16 provided strong evidence against this null hypothesis, indicating statistically significant results. With a Moran’s Index of 0.3437, the analysis revealed positive spatial autocorrelation, suggesting that regions with high levels of under-nutrition tended to cluster together, as did regions with low levels. This observation was further corroborated by a Z-score of 6.28, indicating that the observed Moran’s Index significantly deviated from what would have been expected under random distribution. The vertical dashed lines on the plot signified critical thresholds for statistical significance, providing additional context for the strength of the observed spatial pattern. Overall, these findings underscored the significant clustering of under-nutrition levels within the dataset, highlighting the need for further investigation into the factors influencing this spatial distribution.

### 3.2 Comparative analysis of models and spatial Bayesian analysis of under-nutrition risk factors

The modeling was conducted using four approaches: GLM, GLMM, ICAR, and BYM models, specifically employing a negative binomial distribution. The results presented in Table 4 highlight the goodness-of-fit measures for model comparison through both the Watanabe-Akaike Information Criterion (WAIC) and Leave-One-Out Cross-Validation (LOO), providing valuable insights into their predictive performance. In the WAIC analysis, CAR BYM stands out as the best fit, featuring the highest expected log pointwise predictive density (elpd_waic) at −14833.8 and the lowest WAIC at 29667.5, indicating a strong balance between goodness of fit and model complexity. Model 2 GLMM follows closely similar pattern, while GLM exhibits the weakest performance, with the lowest elpd_waic of −15037.4 and the highest WAIC of 30074.8. This means the GLMM’s performance was not far behind the best-performing model, CAR BYM, it still fell short of its overall effectiveness. The LOO results further confirm the superiority of CAR BYM, which again achieves the highest expected log pointwise predictive density (elpd_loo) at −14833.8 and the lowest LOO Information Criterion (looic) at 29667.6, reflecting its exceptional predictive capability.

**Table 4:** Watanabe-Akaike Information Criterion (WAIC) and Leave-One-Out Cross-Validation (LOO).

Watanabe-Akaike information criterion (WAIC)								
Description	Model 1 GLM		Model 2 GLMM		Model 3 ICAR		Model 4 CAR BYM	
	Estimate	SE	Estimate	SE	Estimate	SE	Estimate	SE
elpd_waic	−15037.4	53.4	−14834.9	54.4	−14837.3	54.5	−14833.8	54.4
p_waic	31.9	1.1	38.4	1.1	38.6	1.1	37.3	1.0
waic	30074.8	106.8	29669.9	108.8	29670.2	108.8	29667.5	108.7

Leave-one-out cross-validation (LOO)								
Description	Model 1 GLM		Model 2 GLMM		Model 3 ICAR		Model 4 BYM	
	Estimate	SE	Estimate	SE	Estimate	SE	Estimate	SE
elpd_loo	−15037.4	53.4	−14835.0	54.4	−14837.3	54.5	−14833.8	54.4
p_loo	32	1.1	35.8	1.1	38.6	1.1	37.3	1.0
Looic	30074.9	106.8	29,670	108.8	29670.2	108.8	29667.6	108.7

Although ICAR has the highest p_loo, indicating greater complexity, it does not perform better in predictive terms compared to CAR BYM. The term p_loo refers to the effective number of parameters in the context of Leave-One-Out Cross-Validation (LOO) for Bayesian models. It provides a measure of model complexity, helping to assess how well a model can be expected to perform on new, unobserved data. Overall, both analyses consistently identify CAR BYM as the best fitted model, establishing it as the most reliable choice for the dataset analyzed.

The CAR BYM model, employing a negative binomial distribution, was selected as the final model due to its superior performance in both WAIC and LOO analyses, and its ability to explicitly account for spatial autocorrelation. The results presented in Table 5 show that the Bayesian CAR BYM model employing a negative Binomial distribution offers a comprehensive analysis of the factors associated with under nutrition among under - five children in Ethiopia. This model is specifically designed to handle count data that exhibit over-dispersion, making it particularly suitable for this type of analysis.

**Table 5:** The estimates of the CAR BYM model.

Parameter	Estimate	Sd	95% CI	Rhat	Bulk_ESS	Tail_ESS
Sd_(Region)*	0.35	0.09	(0.22, 0.58)	1.00	1,553	1,231
Sd_(Cluster)*	0.37	0.14	(0.1, 0.64)	1.01	1,410	973

Regression coefficients (fixed effect)						
Parameter	Estimate	Sd	95% CI	Rhat	Bulk_ESS	Tail_ESS
Intercept*	−2.56	0.11	(2.4, 2.77)	1.01	3,267	2,683
Mother age	−0.00	0.00	(−0.00, 0.00)	1.00	1884	2,427
Mass media (ref = No)						
Yes*	−0.04	0.03	(−0.1, 0.02)	1.00	2,311	2,760
Age of household head	0.00	0.00	(−0.00, −0.00)	1.00	4,283	3,044
Breastfeed (ref = No)						
Yes*	−0.24	0.03	(−0.3, −0.18)	1.00	2,223	2,671
BMI	−0.00	0.00	(−0.00, −0.00)	1.00	3,715	2,791
Water source (ref = Improved)						
Unimproved*	0.12	0.04	(0.04, 0.2)	1.00	2,423	2,591
Toilet facility (ref = improved)						
Unimproved*	0.07	0.03	(0.01, 0.13)	1.00	2,339	2,561
Cooking type (ref = modern)						
Traditional*	0.09	0.02	(0.04, 0.13)	1.00	3,166	3,024
Delivery place (ref = Home)						
Hospital/health center	−0.02	0.01	(−0.04, −0.00)	1.00	2,398	2,234
Residence (ref = rural)						
Urban*	−0.08	0.03	(−0.14, −0.02)	1.00	2,316	2,578
Mother’s education (ref = No edu.)						
Primary*	−0.12	0.02	(−0.16, −0.08)	1.00	3,836	3,342
Secondary*	−0.30	0.04	(−0.38, −0.23)	1.00	3,535	2,867
Higher*	−0.31	0.05	(−0.41, −0.21)	1.00	4,429	3,849
Wealth Index (ref = Poorest)						
Poorer*	−0.08	0.03	(−0.14, −0.02)	1.00	3,023	2,665
Middle*	−0.17	0.02	(−0.22, −0.12)	1.00	3,051	2,775
Rich*	−0.22	0.02	(−0.26, −0.17)	1.00	3,120	2,891
Richest*	−0.47	0.03	(−0.51, −0.40)	1.00	3,021	3,338
Sex of household (ref = Female)						
Male	−0.02	0.01	(−0.04, 0.01)	1.00	1768	2,133

The reference category is denoted as ref. The significant categories are indicated by **∗**, identified by the non-inclusion of zero in the confidence interval (95%CI).

The multilevel hyperparameters given in Table 5 reveal significant variability in under-nutrition rates across both regions and within regions. The sd_(Region) of 0.35 indicates substantial variation in under-nutrition levels between different regions in Ethiopia, even after accounting for the measured household-level factors. Furthermore, the sd_(cluster) of 0.37 highlights significant variability in under-nutrition rates within regions at the sub-cluster level (likely representing districts). These findings underscore the importance of considering both regional and sub-regional factors when designing and implementing interventions to address child under-nutrition in Ethiopia.

The result of spatial modeling in Table 5 can be expressed in the following regression (Equation 29):


(29) $$\begin{array}{c} \;\log(\mu )=2.56-0.04*\text{Mass Medi}a_{\mathrm{yes}}-0.24*{\text{Breastfeed}}_{\mathrm{yes}}\\ +0.12*{\text{water source}}_{\text{unimproved}}+0.07*{\text{toilet}}_{\text{unimproved}}\\ +0.09*\text{cookin}g_{\text{unimroved}}-0.08*{\text{Residence}}_{\text{urban}}\\ -0.12*{\text{Mother education}}_{\text{primary}}\\ -0.3*{\text{Mether education}}_{\text{secondary}}\\ -0.31*{\text{Mether education}}_{\text{higher}}-0.08*{\text{wealth}}_{\text{poorer}}\\ -0.17*{\text{wealth}}_{\text{middle}}-0.22*{\text{wealth}}_{\text{rich}}\\ -0.47*{\text{wealth}}_{\text{richest}}+\Phi_{k}+u_{k} \end{array}$$

When interpreting coefficients from regression models, particularly in the context of count data or logistic regression, the original scale is crucial for understanding the effects of predictors. The appropriate formula for interpretation is:


$$\text{orginal scale}=e^{\text{Coeffient}}-1$$

This formula provides the percentage change in the expected count of the response variable relative to a baseline. It allows researchers to convey the effects of predictor variables in a more intuitive manner, facilitating understanding of how changes in predictors, such as wealth or education, influence outcomes like child nutrition.

Using the formula $\text{orginal scale}=e^{\text{Coeffient}}$ alone offers a multiplicative effect but lacks the contextual clarity of percentage change, making it less useful for practical interpretation. Thus, employing $\text{orginal scale}=e^{\text{Coeffient}}-1$ is preferred for articulating the impact of predictors in a way that is easily understood by stakeholders and policymakers (44).

The CAR BYM model used in this research is spatial Bayes regression using log link function. So the model values presented in Table 5 need to be converted to its original value. To return the model coefficients to their original scale and interpret them, we need to exponentiate the coefficients derived from the log link function used in the negative binomial regression model. This process transforms the coefficients from log counts to counts, allowing for easier interpretation. This can be done using the Equation (18):


$$\text{orginal scale}=e^{\text{Coeffient}}-1$$

Examining the regression coefficients provides valuable insights into the predictors influencing under-nutrition among children. The model results presented in Table 5 indicate an intercept value of 2.56, reflecting the expected log count of undernourished children under five when all predictor variables are at zero (reference levels for categorical predictors). This translates to an estimated count of approximately 13 undernourished children at baseline cluster levels. Access to mass media for mothers has a coefficient of −0.04, suggesting that such access is associated with a 4% lower expected count of under-nutrition in their children compared to those without access holding other factors constant. Notably, breastfeeding practice emerges as a significant protective factor, with a coefficient of −0.24, leading to a 21.3% reduction in the expected count of undernourished children compared to non-breastfed practice holding other factors constant.

Conversely, the coefficient for unimproved water sources is 0.12, indicating that households with unimproved water sources face a 12.7% higher expected count of under-nutrition. Similarly, the coefficient for unimproved toilet facilities is 0.07, suggesting that such household experience a 7.3% higher expected count compared to those with improved facilities holding other factors constant. Additionally, households with traditional cooking practice, with a coefficient of 0.09, are associated with a 9.4% higher expected count of under-nutrition compared to modern cooking methods.

Maternal education plays a crucial role; households with mothers who have primary, secondary, and higher education experience expected counts of under-nutrition reduced by 11.3, 25.9, and 26.4%, respectively compared to those with no education. This highlights the importance of educational initiatives aimed at improving child health outcomes. Urban residence also offers a protective effect, with households living in urban areas showing a 7.7% lower expected count of under-nutrition compared to their rural counterparts.

Wealth significantly impacts nutritional status, with households identified as poorer, middle-income, rich, and richest categories experiencing reductions in expected under-nutrition counts of 7.7, 15.5, 19.7, and 39.8%, respectively compared to those from the poorest households. These findings underscore the urgent need to address socio-economic and environmental conditions alongside health practices to effectively combat under-nutrition in this vulnerable population. This analysis provides valuable insights into the factors associated with under-nutrition among under-five children in Ethiopia. Maternal education, household wealth, breastfeeding practices, access to clean water and sanitation, and place of residence are significant predictors of under-nutrition.

This analysis, primarily focused on the results from the CAR BYM model, provides valuable insights into the spatial distribution of under-nutrition and the associated risk factors among under-five children in Ethiopia.

### 3.3 Model diagnosis

The Potential Scale Reduction Factor (Rhat) values are all near one, and the effective sample sizes (Bulk_ESS and Tail_ESS) are large, as shown in Table 5. This indicates that the Markov Chain Monte Carlo (MCMC) chains have converged effectively and that the estimates are stable.

**Figure 4:**
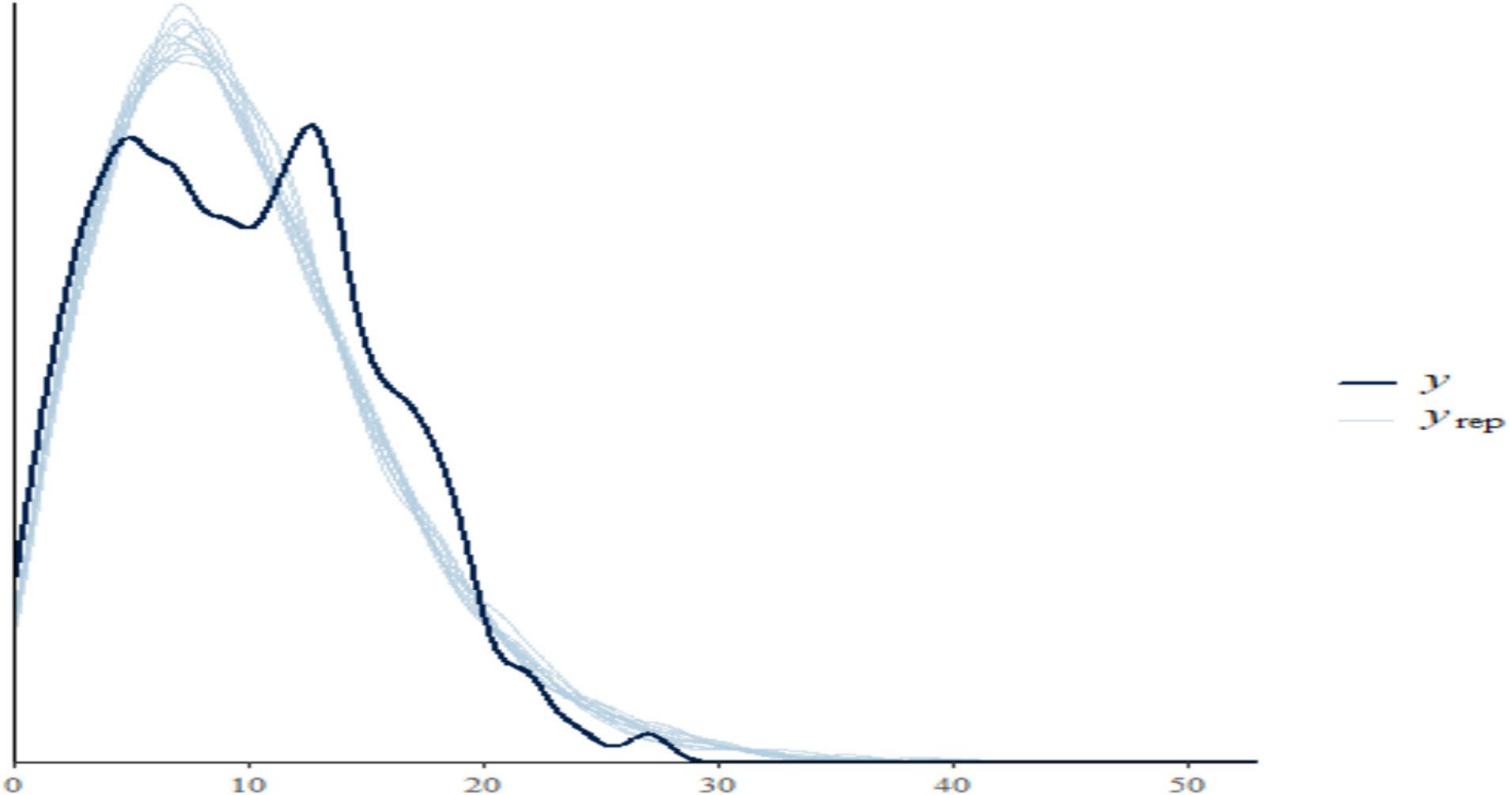
The posterior predictive checks plot of undernourished children.

The posterior predictive checks plot presented in Figure 4 visually compares the observed data with the replicated data generated by the model. The darker line represents the distribution of the observed counts of undernourished individuals, while the lighter lines depict the distributions of counts produced from the model using the posterior predictive distribution. A close alignment between the light lines (y_rep) and the dark line (y) indicates that the model fits the observed data well; specifically, if the replicated data captures the general shape and range of the observed data, it suggests effective modeling of the underlying distribution. In this plot, both distributions exhibit similar shapes, with a peak at lower counts and a gradual decline as counts increase, indicating that the model successfully captures the data’s characteristics. The spread of the light lines reflects the variability in the replicated data; if they are tightly clustered around the observed data, it indicates reliable predictions. Conversely, significant discrepancies would suggest potential model inadequacies. Overall, the close alignment between the distributions of observed and replicated data suggests that the model effectively predicts the counts of undernourished individuals based on the included predictors.

### 3.4 Mapping relative risk using the CAR BYM model

**Figure 5:**
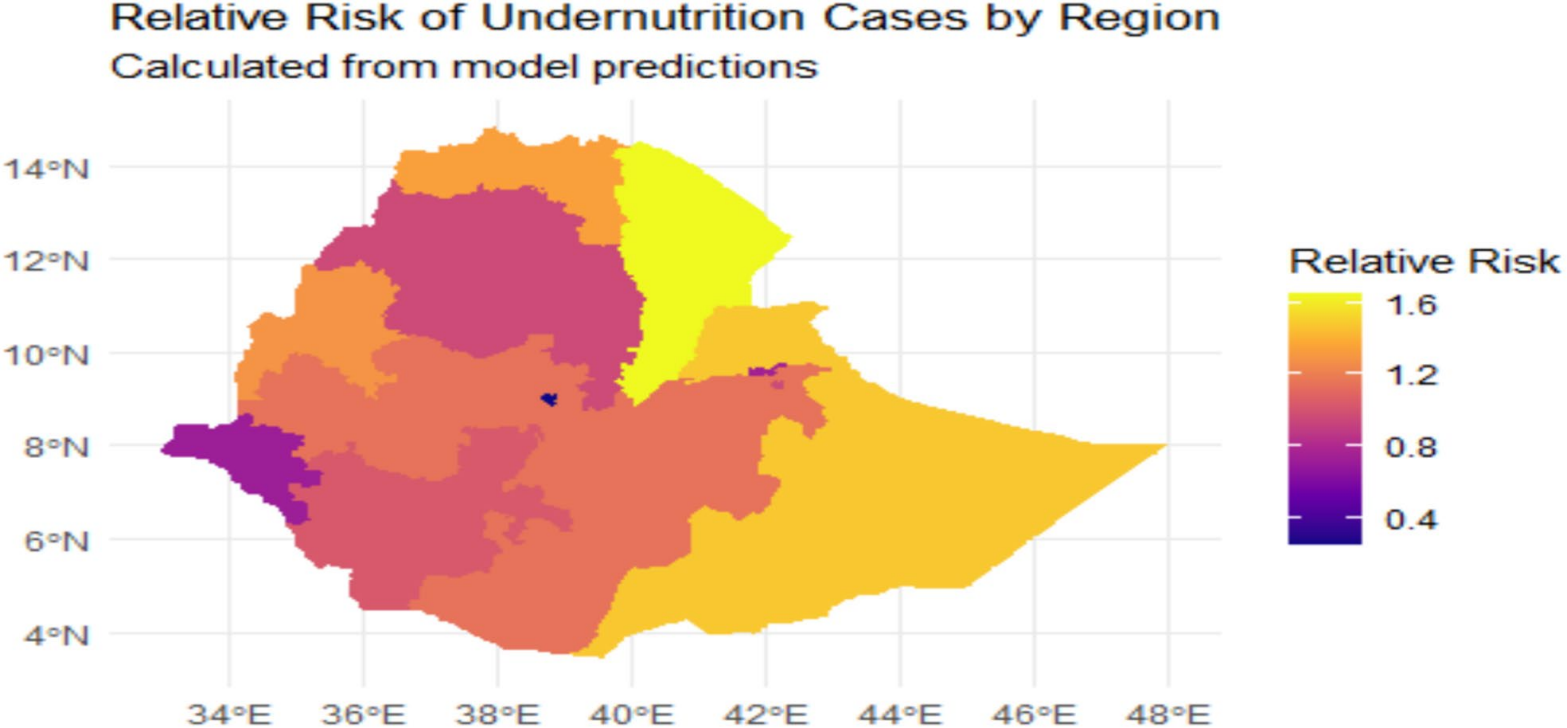
Relative risk map of under nutrition cases in regions, Ethiopia 2019.

We can visualize relative risks, which are composed of both spatially structured and unstructured components. This representation provides crucial information regarding the future probability of under nutrition cases. The probability map illustrating these relative risks is shown in Figure 5.

The relative risk (RR) of under nutrition in Ethiopia, as illustrated by the map given in Figure 5, quantifies the likelihood of under nutrition occurring in various regions. This can be expressed in percentage terms, where an RR of one represents a baseline risk (0% increase), while an RR of 1.2 indicates a 20% higher risk, an RR of 1.5 signifies a 50% higher risk, and an RR of 1.6 corresponds to a 60% increase in risk compared to the baseline. This framework allows for a clearer understanding of the severity of under nutrition across different areas. Regions identified as having a high relative risk (RR) exceeding 1.5 include the Somali Region, which exhibits the highest risk. This indicates a 50 to 60% increase in the likelihood of under-nutrition cases compared to the baseline risk. Specifically, an RR of 1.5 suggests that the probability of under-nutrition in this region is 50% higher than in areas with an RR of one, which represents the baseline risk. This framework highlights the urgent need for targeted interventions in regions with elevated risk levels. The Afar Region also reflects significant risk, as indicated by its relative risk values. Additionally, certain areas within the Oromia Region show high RR values. In contrast, regions categorized as having a moderate relative risk (RR between one and 1.5) include the Amhara Region, identified as areas experiencing a 20 to 50% increase in under nutrition. The Tigray Region similarly shows moderate risk. Parts of the Southern Nations, Nationalities, and Peoples’ Region (SNNPR) are also classified in this category. Regions classified with a low relative risk (RR below one) include Addis Ababa, where improved access to nutrition and healthcare resources could be considered as contributes to lower risk levels. Similarly, the Gambela and Benishangul-Gumuz Regions exhibit reduced relative risk.

## 4 Discussion

This study highlights the critical issue of under-nutrition among children under five in Ethiopia, revealing the multifaceted factors that contribute to this pervasive public health challenge. The results derived from the Bayesian Conditional Autoregressive (CAR) Besag-York-Mollié (BYM) model, which effectively addresses over-dispersion in count data, provide a nuanced understanding of the predictors influencing under-nutrition rates across different regions.

The results highlight the significant impact of some socioeconomic factors on child nutrition. The negative correlation between household wealth and under-nutrition rates is particularly noteworthy. As families transition from lower to higher wealth categories, the risk of under-nutrition diminishes significantly, with the wealthiest households demonstrating an impressive 37% reduction in expected cases. This aligns with existing literature that emphasizes the importance of economic stability in ensuring access to nutritious foods and healthcare services (5, 6). The pronounced disparities between urban and rural households highlight the urgent need for targeted interventions that address food insecurity and resource limitations in rural areas (7).

Maternal education emerged as another vital determinant of child nutrition. The model indicates that each level of maternal education correlates with significant reductions in under-nutrition rates. Specifically, primary, secondary, and higher education levels are associated with decreases of 11.3, 25.9, and 26.4%, respectively. These findings support previous research suggesting that educated mothers are more likely to adopt health-promoting behaviors, understand nutritional requirements, and utilize available healthcare resources (8, 9). Thus, enhancing maternal education through targeted programs and interventions can be instrumental in improving child nutritional status.

Breastfeeding practices also play a crucial role in child nutrition, with the model indicating a 21.3% reduction in the expected count of undernourished children compared to non-breastfed. This suggests ongoing challenges in adhering to optimal breastfeeding guidelines (10). Educational initiatives aimed at promoting exclusive breastfeeding and addressing common barriers faced by mothers are essential for improving nutritional status among young children.

The analysis reveals the importance of environmental factors, such as access to improved water and sanitation. The positive coefficient for unimproved water sources suggests that households with unimproved water sources face a 12.7% higher expected count of under-nutrition. Similarly, the positive coefficient for unimproved toilet facilities indicates that such households experience higher expected count of under-nutrition. Furthermore, the positive association between unimproved toilet facilities and under-nutrition rates underscores the critical need for comprehensive sanitation and hygiene programs (12, 13). Improving water quality and sanitation can mitigate the risk of diseases that exacerbate malnutrition, particularly in vulnerable populations. Additionally, the analysis reveals that traditional cooking practices are significantly associated with higher rates of under-nutrition. This finding aligns with existing literature that emphasizes the importance of food preparation methods in ensuring adequate nutrition for children (17). Addressing cooking practices through community education and resources could be a vital area for intervention, particularly in regions where traditional methods prevail.

The spatial modeling conducted using the CAR BYM model offers a nuanced understanding of the relative risk of under-nutrition across Ethiopia’s regions. High-risk areas, such as Somali, Afar, and parts of Oromia, are identified as priority regions necessitating immediate, targeted interventions. In contrast, regions like Addis Ababa, Gambela, and Benishangul-Gumuz demonstrate relatively lower risk. These lower-risk areas could serve as valuable references, offering insights into effective strategies and practices that might be adapted to mitigate under-nutrition in the high-risk regions.

## 5 Conclusion

This study aimed to model and map the number of under-nutrition cases across various regions in Ethiopia, employing four distinct modeling approaches: Generalized Linear Models (GLM), Generalized Linear Mixed Models (GLMM), Intrinsic Conditional Autoregressive (ICAR) models, and Conditional Autoregressive Besag-York-Mollié (CAR BYM) models. The initial GLM and GLMM served as baseline models, allowing for the assessment of the impact of incorporating hierarchical and spatial structures. However, the spatial Bayesian models, ICAR and particularly CAR BYM, which explicitly accounted for spatial autocorrelation, demonstrated superior performance in terms of model fit and predictive accuracy, as evidenced by the WAIC and LOO criteria.

The CAR BYM model, which incorporated both fixed effects and spatially structured random effects, was identified as the most robust model for mapping under-nutrition prevalence. The results from this model revealed that maternal education, breastfeeding practices, access to clean water and sanitation facilities, cooking practices, household wealth, place of residence, and regional disparities are significant covariates influencing under-nutrition among children under five in Ethiopia. The spatial analysis using the CAR BYM model further identified high-risk regions, such as Somali, Afar, and parts of Oromia, emphasizing the urgent need for targeted interventions in these areas.

The findings of this research highlight the importance of utilizing spatial Bayesian models, like the CAR BYM, for understanding and addressing the complex spatial patterns of under-nutrition. By highlighting the impact of various socio-environmental factors on under-nutrition prevalence, the study provides greater understanding to policymakers and public health practitioners to develop effective strategies for improving child nutrition outcomes. It is recommended that, among others, health education and income-generating initiatives implemented through targeted interventions focusing on high-risk regions identified through spatial analysis will improve child nutrition status of the country.

The comparative analysis of the four models underscores the critical role of spatial modeling in accurately capturing and predicting under-nutrition prevalence. The CAR BYM model’s ability to account for spatial dependencies and hierarchical data structures resulted in a more nuanced and accurate representation of under-nutrition patterns compared to the non-spatial GLM and the partially spatial GLMM. This study validates the utility of spatial Bayesian models in public health research, particularly in contexts where geographical factors significantly influence health outcomes.

## Data Availability

The original contributions presented in the study are included in the article/supplementary material, further inquiries can be directed to the corresponding author/s.
